# Levelling off of prevalence of obesity in the adult population of Sweden between 2000/01 and 2004/05

**DOI:** 10.1186/1471-2458-10-119

**Published:** 2010-03-09

**Authors:** Jan Sundquist, Sven-Erik Johansson, Kristina Sundquist

**Affiliations:** 1 Deparment of Clinical Science, Center for Primary Health Care Research, Lund University, Malmö, Sweden; 2 Stanford Prevention Research Center, Stanford University, Palo Alto, California, USA; 3 Department of Neurobiology, Health Care Sciences and Society/Centre for Family and Community Medicine, Karolinska Institutet, Stockholm, Sweden

## Abstract

**Background:**

The escalating global epidemic of obesity is of worldwide concern because of its association with several chronic diseases and premature mortality. Some subgroups seem to be more affected than others. The aim of this study was to examine whether the mean BMI (adjusted for age) and the prevalence of obesity (adjusted for all the explanatory variables) changed between 2000/01 and 2004/05 in different subgroups of the Swedish population.

**Methods:**

This study compared two cross-sectional, nationwide random samples of persons aged 16 to 84 years: the first from 2000/01 (5515 men, 5838 women) and the second from 2004/05 (4681 men, 4821 women). After stratification by gender, a logistic regression model was applied to analyse possible changes in mean BMI and the prevalence of obesity between 2000/01 and 2004/05.

**Results:**

Total mean BMI remained almost unchanged between 2000/01 and 2004/05 for both men and women. The prevalence of obesity increased slightly in both men and women, but not significantly (from 9.7 to 10.8% and from 9.6 to 10.2%, respectively). The prevalence of obesity in 2004/05 was especially high in some subgroups: men aged 45-54 (14.3%) or 55-64 (16.5%), women aged 65-74 (15.9%) or 75-84 (16.8%), men and women of middle educational level (15.6% and 14.4%, respectively), male former smokers (13.4%), and men from small towns or rural areas (13.1%).

**Conclusions:**

Although the mean BMI and obesity were almost unchanged in the Swedish adult population between 2000/01 and 2004/05, obesity levels in Sweden remained unacceptably high, especially in certain subgroups. Primary and secondary intervention actions should strive to decrease the prevalence of obesity in Sweden.

## Background

The prevalence of obesity has reached epidemic proportions in developed countries and is a growing problem in developing countries. The consequences of obesity include dyslipidaemia, [1] insulin resistance, [1] the metabolic syndrome, [1] low-grade inflammation, [2,3] diabetes, [1] cancer, [4] accelerated ageing, [5,6] musculoskeletal disorders, [7] sleep apnoea [7] and alterations in growth factors and other hormones that are important for the development of arteriosclerosis [1-3].

Disturbingly, studies from Sweden and elsewhere in the developed world indicate that the prevalence of obesity continues to rise [8-17]. The high prevalence of obesity and its negative health effects give the disease a ranking as one of the foremost problems confronting health authorities worldwide.

The most recent studies on obesity and body mass index (BMI) trends in the Swedish population trace developments up to 2000/01. We set out to update the available information on these trends in total and in a variety of subgroups of the Swedish population to fill a gap in the existing data. The updated data can be used to assess progress or a lack thereof in stopping the spread of the obesity epidemic in Sweden, and to help target interventions to where they are needed the most, i.e., to the most vulnerable segments of the population. For example, a recent study demonstrated that the magnitude of the obesity problem is particularly high in urban deprived neighbourhoods in Stockholm County, Sweden, where one third of the sample in a deprived neighbourhood was classified as overall obese. After adjusting for age, Middle Eastern immigrants had a 3.10 higher odds of being obese than their Swedish-born counterparts [18].

In a previous study on BMI and obesity trends in Sweden from 1996/97 to 2000/01, we found that during that short study period, BMI had increased by 0.4 units in men and women, and the prevalence of obesity had gone up by 10%. There was a high prevalence of obesity among persons between age 55 and 74 years, men of middle educational level, women of low educational level, former smokers, and men and women born in Finland [8]. These results were consistent with the results of other studies on obesity in the Swedish population [19,20].

The current study is based on two simple random samples, representative of the entire Swedish non-institutionalized population, aged 16-84 years. The samples were drawn from the Total Population Register and included both Swedish-born and foreign-born men and women representative of different socioeconomic groups.

The aim of this study was to examine whether mean BMI (adjusted for age) and the prevalence of obesity (adjusted for all explanatory variables) changed in Sweden between 2000/01 and 2004/05, both in the total and in different subgroups of the population. The following explanatory variables were included: education, smoking habits, urbanization and immigrant status.

## Methods

### The Swedish Annual Level of Living Survey

The Swedish Annual Level of Living Survey (SALLS) was the source of the information analysed in this study. The SALLS has been conducted yearly since 1974 by Statistics Sweden, the Swedish governmental statistics agency. SALLS is a nationally representative, simple cross-sectional random sample of adult, non-institutionalized persons aged 16-84 years, taken from the Total Population Register that includes the entire population of Sweden. The main objective of the SALLS is to provide a source of reliable data on which to base public debate and sociopolitical reforms. The statistics generated are expected to provide information on living conditions, on relationships between problems in different social areas and on differences between various subgroups of the population.

Since 1979, there have been four main themes in the SALLS: social relations, work, health and the physical environment. Every annual survey includes a number of indicators for these four themes. Certain questions about health, employment, economic resources, working environment, education and housing are repeated every year in order to provide consistent information on important background variables, e.g., self-reported health, socioeconomic conditions and family type. Other questions, which are not repeated annually, provide information that makes it possible to follow changes in some selected fields, such as lifestyle factors. Professional interviewers from Statistics Sweden conduct the interviews one-on-one, usually at the respondents' homes [21]. The data are not publicly available and the use and analysis of the data need permission from Statistics Sweden.

### Outcome variables

The outcome variables included *body mass index (BMI) *and *obesity*. BMI was calculated as weight(kg)/height^2^(m^2^), and obesity was defined as BMI ≥ 30 and overweight as 25 ≤ BMI < 30. In accordance with recommendations from the World Health Organization and the National Heart, Lung and Blood Institute Expert Panel, the same criteria for obesity were used for both women and men [22,23]. BMI was also analysed as a continuous variable. Weight and height were self-reported.

### Explanatory variables

We chose to include six explanatory variables for which previous studies have suggested an association with BMI and obesity: sex, [24] age, [8] educational level, [8,17] smoking habits, [25] urbanization [26] and immigrant status [8,27].

*Sex:* Separate analyses were undertaken for men and women.

*Age:* Age was categorized into the following groups: 16-24, 25-34, 35-44, 45-54, 55-64, 65-74 and 75-84.

*Educational level:* Educational level was divided into compulsory school or less (≤ 9 years); practical high school, i.e., vocational school (10-11 years); and theoretical high school and/or college (≥ 12 years).

*Smoking habits:* Respondents were divided into three groups based on current tobacco consumption: (1) those who had never smoked, (2) former smokers (regardless of when they stopped smoking) and (3) daily smokers.

*Urbanization:* This variable was broken down into three categories: large cities (the three largest cities in Sweden - Stockholm, Gothenburg, and Malmo), medium-sized towns (> 90,000), and small towns (27,000-90,000) or rural areas.

*Immigrant status *was defined as follows: (1) Swedish-born people (both parents born in Sweden), (2) second-generation immigrants (with at least one parent born abroad) and (3) first-generation immigrants. The third group was divided into two subgroups: labour immigrants and refugees (immigrants born outside Europe).

### Statistical analysis

The distribution of mean BMI by the explanatory variables was calculated. The differences in BMI between the two periods were age-adjusted for each category (subgroup) of the different explanatory variables.

Persons with missing values in the variables weight and height were excluded when calculating mean BMI. However, when calculating overweight and obesity prevalence rates, those with missing values were included in the normal BMI group.

The prevalence rates were estimated by employing individual weights, which were calculated by post-stratification using the variables sex, age, civil status (married or not married), and geographic region (six regions), resulting in approximately 80 strata. The weight was obtained by dividing the population size in each stratum, obtained from the Total Population Register for the year of the data collection, by the corresponding sample size. The sum of the weights adds up to the population size. We applied these weights in all estimations.

A logistic regression model (adjusted for all explanatory variables) was applied to test the change in obesity between the two periods in different subgroups. The results are shown as odds ratios (ORs) with 95% confidence intervals (CIs). The overall change in OR is estimated separately by sex.

The SAS software package was used in the statistical analyses [28].

## Results

The distribution of persons in the sample in percentages by the explanatory variables, the mean BMI by the explanatory variables, and tests of change in the mean BMI (p value) between 2000/01 and 2004/05 are shown in Table 1 (men) and Table 2 (women). The test of change is shown for each row (adjusted for age).

**Table 1:** Weighted distribution (%) of the explanatory variables and age-adjusted mean BMI of men in 2000/01 and 2004/05 by the explanatory variables, and results of a test of change (p value) between the two periods.

		2000/01 (n = 5515)		2004/05 (n = 4681)		P values for test of change
			
Explanatory variable	Category (subgroup)	Distribution (%)	BMI mean	Distribution (%)	BMI mean	
Totals	16-84		25.4		25.5	Ns
Age	16-24	13.5	23.1	14.1	23.0	Ns
	25-34	18.1	24.8	16.4	24.9	Ns
	35-44	17.9	25.8	18.7	26.2	0.013
	45-54	18.2	26.1	16.3	26.4	0.04
	55-64	15.4	26.3	17.8	26.5	Ns
	65-74	10.1	26.3	10.2	26.1	Ns
	75-84	6.8	25.4	6.5	25.2	Ns
Educational level (years)	≤9	25.2	25.5	23.5	25.6	Ns
	10-11	27.7	25.9	25.0	26.3	0.010
	≥12	47.1	25.0	51.5	25.2	0.015
Smoking	Never	48.4	25.3	51.7	25.4	Ns
	Former	34.2	25.7	34.1	25.8	Ns
	Daily	17.4	24.8	14.2	25.3	0.009
Urbanization^1^	1	34.7	25.1	35.3	25.2	Ns
	2	36.1	25.5	36.2	25.6	Ns
	3	29.2	25.5	28.5	25.8	0.02
Immigrant	Sweden	83.3	25.3	80.9	25.4	Ns
Status	Second-generation immigrants	6.1	25.5	6.4	25.9	Ns
	Labour immigrants	4.8	25.6	5.6	25.8	Ns
	Refugees	5.8	25.6	7.1	25.7	Ns

All persons in the sample were between 16 and 85 years old.

^1 ^(1) The three largest cities in Sweden, (2) medium-sized towns (> 90,000) and (3) small towns (27,000-90,000) or rural areas.

**Table 2:** Weighted distribution (%) of the explanatory variables and age-adjusted mean BMI of women in 2000/01 and 2004/05 by the explanatory variables, and results of a test of change (p value) between the two periods.

		2000/01 (n = 5838)		2004/05 (4821)		P values for test of change
			
Explanatory variable	Category (subgroup)	Distribution (%)	BMI mean	Distribution (%)	BMI mean	
Totals	16-84		24.4		24.3	Ns
Age	16-24	12.6	21.9	13.4	21.8	Ns
	25-34	17.0	23.4	16.4	23.5	Ns
	35-44	16.9	24.3	17.4	24.5	Ns
	45-54	17.3	24.7	17.1	24.7	Ns
	55-64	15.3	25.8	16.4	25.4	0.02
	65-74	11.3	25.7	10.9	25.8	Ns
	75-84	9.6	25.2	8.4	25.3	Ns
Educational level (years)	≤9	26.4	24.9	23.1	24.7	Ns
	10-11	30.6	24.6	25.1	24.9	0.045
	≥12	43.0	24.0	51.8	24.1	Ns
Smoking	Never	54.5	24.4	54.2	24.3	Ns
	Former	25.1	24.7	28.2	24.6	Ns
	Daily	20.4	23.9	17.6	24.0	Ns
Urbanization^2^	1	35.7	24.0	36.2	24.0	Ns
	2	35.8	24.5	36.2	24.5	Ns
	3	28.5	24.9	27.6	24.8	Ns
Immigrant	Sweden	82.1	24.3	80.3	24.3	Ns
Status	Second-generation immigrants	6.3	24.5	6.7	24.7	Ns
	Labour immigrants	5.8	25.2	5.7	24.9	Ns
	Refugees	5.8	25.3	7.2	25.1	Ns

All persons in the sample were between 16 and 84 years old.

^1 ^(1) The three largest cities in Sweden, (2) medium-sized towns (> 90,000) and (3) small towns (27,000-90,000) or rural areas.

The mean BMI did not change significantly (from 25.4 to 25.5) during the study period among men. Table 1 shows that mean BMI increased in some subgroups of men, for example, men aged 35-44 or 45-54, men of middle or high educational level and men who smoked daily. The mean BMI of men in the third urbanization group increased slightly.

The mean BMI did not change significantly among women between 2000/01 and 2004/05. Table 2 shows that BMI increased in only one subgroup of women, i.e., those of middle educational level. BMI decreased in one group of women, those aged 55-64.

The prevalence of overweight and obesity and tests of change in the prevalence of obesity between 2000/01 and 2004/05 are presented in Table 3 (men) and Table 4 (women). The models are adjusted for all the explanatory variables. For men of all ages, the prevalence of obesity increased from 9.7% in 2000/01 to 10.8% in 2004/05, but the increase was not statistically significant. Table 3 shows a statistically significant increase in the prevalence of obesity in men aged 35-44 and men with a high educational level.

**Table 3:** Weighted prevalence (%) of overweight (25≤BMI<30) and obesity (BMI≥30) by the explanatory variables for men in the two periods, and test of change (logistic regression) in obesity adjusted for age, education, smoking habits, urbanization and immigrant status.

		2000/01		2004/05		Test of change in obesity prevalence
		
Explanatory variable	Category (subgroup)	Overweight 25≤BMI<30	Obesity BMI≥30	Overweight 25≤BMI<30	Obesity BMI≥30	Odds ratio (95% confidence interval)
Totals	16-84	40.9	9.7	40.6	10.8	1.13 (0.99-1.29)
Age	16-24	17.8	3.3	17.2	3.4	0.98 (0.56-1.73)
	25-34	33.3	7.9	33.8	6.2	0.90 (0.62-1.30)
	35-44	45.9	9.3	44.6	12.4	1.45 (1.08-1.96)*
	45-54	48.3	11.8	49.5	14.3	1.25 (0.94-1.66)
	55-64	49.5	13.2	49.4	16.5	1.30 (0.99-1.70)
	65-74	47.9	14.2	46.2	12.0	0.83 (0.58-1.20)
	75-84	42.9	7.9	41.9	5.6	0.70 (0.38-1.30)
Educational level (years)	≤9	38.9	10.7	36.2	10.8	1.06 (0.82-1.38)
	10-11	45.6	14.1	47.8	15.6	1.05 (0.85-1.30)
	≥12	38.2	6.5	38.6	8.2	1.29 (1.04-1.60)*
Smoking	Never	37.5	7.3	37.2	8.6	1.22 (0.99-1.49)
	Former	46.2	13.5	44.5	13.4	1.00 (0.82-1.21)
	Daily	37.4	8.4	41.7	11.6	1.36 (0.97-1.90)
Urbanization^1^	1	38.3	8.0	38.4	8.5	1.09 (0.85-1.40)
	2	42.5	10.0	40.6	10.9	1.17 (0.95-1.45)
	3	40.4	11.1	42.2	13.1	1.13 (0.91-1.41)
Immigrant	Sweden	40.7	10.6	41.0	10.1	1.10 (0.95-1.27)
Status	Second-generation immigrants	36.6	10.1	35.2	12.3	1.35 (0.82-2.24)
	Labour immigrants	49.0	12.3	41.9	13.7	1.27 (0.75-2.15)
	Refugees	40.7	7.9	41.2	9.3	1.25 (0.72-2.18)

All persons in the sample were between 16 and 84 years old.

^1 ^(1) The three largest cities in Sweden, (2) medium-sized towns (> 90,000) and (3) small towns (27,000-90,000) or rural areas.

The overall OR is estimated separately

**Table 4:** Weighted prevalence (%) of overweight (25≤BMI<30) and obesity (BMI≥30) by the explanatory variables for women in the two periods, and test of change (logistic regression) in obesity adjusted for age, education, smoking habits, urbanization and immigrant status.

		2000/01		2004/05		Test of change in obesity prevalence
		
Explanatory Variable	Category (subgroup)	Overweight 25≤BMI<30	Obesity BMI≥30	Overweight 25≤BMI<30	Obesity BMI≥30	Odds ratio (95% confidence interval)
Totals	16-84	27.3	9.6	25.9	10.2	1.09 (0.95-1.24)
Age	16-24	11.9	1.6	10.2	2.6	1.52 (0.71-3.23)
	25-34	18.8	6.9	17.0	6.8	1.16 (0.80-1.67)
	35-44	24.6	9.2	23.0	11.1	1.22 (0.90-1.65)
	45-54	30.3	9.2	30.1	8.8	1.00 (0.72-1.38)
	55-64	35.9	14.4	35.7	13.0	0.90 (0.68-1.19)
	65-74	39.3	14.3	37.2	15.9	1.11 (0.80-1.54)
	75-84	34.8	13.1	32.4	16.8	1.19 (0.81-1.74)
Educational Level (years)	≤9	28.6	12.5	25.8	11.2	0.85 (0.66-1.09)
	10-11	31.7	9.9	31.0	14.4	1.46 (1.17-1.82)*
	≥12	21.9	6.9	21.9	7.1	0.99 (0.80-1.23)
Smoking	Never	26.3	8.5	24.6	8.9	1.10 (0.91-1.32)
	Former	28.7	12.3	26.8	12.0	1.00 (0.80-1.26)
	Daily	25.2	7.8	24.2	9.6	1.23 (0.90-1.68)
Urbanization^2^	1	23.2	7.9	22.3	8.1	0.99 (0.78-1.27)
	2	26.6	9.6	25.8	9.6	1.03 (0.83-1.29)
	3	31.1	10.7	28.0	12.5	1.23 (0.99-1.54)
Immigrant	Sweden	27.1	9.2	25.9	9.9	1.09 (0.94-1.26)
Status	Second-generation immigrants	24.5	8.2	22.5	8.7	1.03 (0.60-1.75)
	Labour immigrants	36.2	14.1	30.6	13.5	0.73 (0.39-1.37)
	Refugees	24.1	11.5	26.2	11.7	0.82 (0.50-1.33)

All persons in the sample were between 16 and 84 years old.

^1 ^(1) The three largest cities in Sweden, (2) medium-sized towns (> 90,000) and (3) small towns (27,000-90,000) or rural areas.

The overall OR is estimated separately.

For women of all ages, there was a slight, although not significant, increase in the prevalence of obesity (from 9.6% to 10.2%) between 2000/01 and 2004/05. Table 4 also shows that the increase in obesity was significant only among women with a middle educational level.

## Discussion

The main finding of this study is that mean BMI and the prevalence of obesity appear to have levelled off in the Swedish adult population between 2000/01 and 2004/05 in both men and women. In three subgroups there were, however, significant increases in the prevalence of obesity, i.e. among men aged 35-44, among men with a high educational level and among women with a middle educational level.

In addition, in some subgroups, the prevalence of obesity was especially high, for example, men aged 45-54 or 55-64, women aged 65-74 or 75-84, men and women with a middle educational level, men and women who were former smokers, men and women in rural areas and male and female labour immigrants.

Our findings of a levelling off trend in the prevalence of obesity in women were in line with the findings of an American study [27] that demonstrated equal prevalence rates of obesity in women in 1999/2000 and 2003/04, although at a much higher level than in Sweden. Most other existing studies from Spain, Canada, Denmark, Portugal, South Australia and Finland show that the prevalence of obesity increased in both men and women between the mid-1990s and the beginning of the 2000s [12,13,15,29,30]., A European review revealed that the prevalence of obesity varies widely from country to country, with higher prevalence rates in Southern, Eastern and Central Europe. In general, the prevalence of obesity appears to be lower in European countries than in the United States [31].

Our finding of a 14.3% prevalence of obesity in men aged 44-54 in 2004/05 agreed with the findings of another recently published Swedish study, i.e. the Gothenburg study of men aged 50. The Gothenburg study population had a prevalence of obesity of 13.8% in 2003 [32].

Another Swedish study has shown that the obesity epidemic among 10-year- old children had levelled off in Stockholm in 2003[33].

We can only speculate about the reasons for the levelling off of mean BMI and the prevalence of obesity in Sweden between 2000/01 and 2004/05. For example, during these four years the Swedish National Institute of Public Health http://www.fhi.se/en/ has emphasized increased physical activity and better eating habits as two of the most significant areas to focus on in order to improve public health in Sweden. Since the turn of the millennium, the Swedish National Institute of Public Health has implemented a number of campaigns to increase physical activity in all age groups. Medical prescription of physical activity for primary health care patients by doctors and other health care workers is one such effort that has received positive reviews from both doctors and patients [34]. The Swedish National Food Administration (NFA), which is responsible for Swedish nutritional recommendations and for dietary recommendations for different groups in the population, has been active in promoting healthy eating habits in Sweden during the study period.

There is also an increasing interest in the media in informing the public about the health benefits of healthy food and physical activity. For example, one of the largest daily newspapers publishes a once-a-week health supplement. All these steps may have been important in raising consciousness regarding diet and physical activity in Sweden.

But public awareness does not necessarily equal to action that is sufficient to counteract the problem of obesity, as evidenced by other countries' experiences. For example, in the U.S. and Canada, serious media and governmental attention has been paid to the problem of obesity, [35] but despite this, obesity prevalence continues to rise in many groups [27,36,37]. Therefore, it appears that the mechanisms behind increasing obesity are complex and may vary by country or culture.

The levelling off of obesity and BMI in Sweden may be due to some factor or factors other than increased awareness. It may be due to factors in Sweden that make it easier to translate increased awareness into action. Or it may be due to the absence in Sweden of factors that exist in other countries that tip the balance in favour of increasing obesity and BMI despite increased public awareness of the problem. Such factors may include a relative lack of walkable neighbourhoods and public transportation options. They may include comparatively low fast food restaurant prices and comparatively large portion sizes. They may include differences in some subgroups of the population, such as increased genetic vulnerability to obesity; lower levels of knowledge about healthy food, physical activity or the consequences of obesity; or varying receptivity to messages about healthy food and physical activity. Other and possibly more complex social, sociopolitical or cultural factors may play a role, or some combination of all these factors may work together to tip the balance in favour of increasing obesity and BMI.

General mechanisms behind obesity and high mean BMI include a poor diet and physical inactivity. A high-fat diet is probably the most important factor in the development of obesity in genetically predisposed individuals [38]. Results from a recently published randomized controlled trial revealed that caloric restriction alone or in combination with physical activity can reduce body weight, fasting insulin, core body temperature, triiodothyronine (T3) levels and damage to DNA, [39] factors associated with the development of arteriosclerosis, diabetes and some types of cancer [1,4]. Additionally, another study has shown that an optimal 6-year calorie-restricted diet in highly motivated individuals reduced fasting insulin, fasting glucose, inflammatory markers and blood pressure [40].

Physical activity also plays an important role in BMI and obesity. A previous study showed that Swedish men who became physically inactive had a higher increase in BMI between 1980-81 and 1988-89 than those who were physically active on a regular basis [41]. Four independent, cross-sectional population surveys (the FINRISK Studies) conducted in Finland between 1982 and 1997 showed that the inverse association of the level of leisure-time physical activity with BMI was significantly strengthened over the 15-year period in both sexes [42]. Other studies have confirmed the negative effect of physical inactivity on BMI, [41] premature mortality in elderly persons [43] and coronary heart disease [44].

This study has some important limitations. Unfortunately, we had no physical activity data on those who participated in the 2000/01 part of the study. Therefore, we were not able to adjust for physical activity in the statistical models. We hypothesized that physical activity levels might be associated with the levelling off of BMI and obesity in Sweden. Thus, we checked for changes in levels of leisure-time physical activity in persons in the sample. These data are available from the SALLS 1996/97 and 2004/05, as both surveys include a question about physical activity. We found that the percentage of subjects in our sample who took part in leisure-time physical activities at least once a week remained high during 2004/05 (60%)compared with 1996/97 (55%) (data not shown). Another limitation is that our outcome measures are based on self-reported assessments of height and weight, which might underestimate the absolute BMI levels and prevalence of obesity because of a self-report bias [45] in the SALLS. Furthermore, socioeconomic differences in the validity of self-report instruments for estimating BMI have been reported in Sweden [46]. However, the level of self-report bias is probably the same for both periods, thus resulting in a correct estimate of change between the two periods. Another limitation is that the non-response rate might result in a further underestimation of the prevalence of obesity. However, the non-response rate in SALLS is relatively low, compared with surveys from many other countries (see below). The non-response rate was also about the same during the two periods of time. Another limitation is that residual confounding probably exists for the educational level variable because individual education cannot be measured precisely and completely [47,48]. For example, the variable 'years of education' does not capture the quality of schooling or literacy levels.

This study also has several strengths. The SALLS is one of the most comprehensive national surveys to date and has been conducted in Sweden for more than thirty years. Unlike many surveys, each SALLS represents a simple random sample, drawn from the Total Population Register, and is therefore representative of the entire Swedish population. The surveys in the present study, mainly conducted at the respondents' homes as face-to-face interviews, have low non-response rates (about 24%) with a minimum of partially missing data.

## Conclusions

The 2000/01 and 2004/05 prevalence estimates presented in this study indicate that the previously observed trend towards increasing obesity and BMI is levelling off in Sweden. However, the prevalence rates of obesity among men and women are still higher than in previous generations, which is of concern because the national burden of chronic diseases in the population is highly associated with obesity in both men and women.

## Competing interests

The authors declare that they have no competing interests.

## Authors' contributions

All authors conceived and designed the study, were involved in the analyses and drafted the manuscript.

## Pre-publication history

The pre-publication history for this paper can be accessed here:

http://www.biomedcentral.com/1471-2458/10/119/prepub
